# Supplementary material for: Identification of a Hydroxygallic Acid Derivative, Zingibroside R1 and a Sterol Lipid as Potential Active Ingredients of Cuscuta chinensis Extract That Has Neuroprotective and Antioxidant Effects in Aged Caenorhabditis elegans
Source: Nutrients. 2022 Oct 9;14(19):4199. doi: 10.3390/nu14194199 (PMC9570774; doi:10.3390/nu14194199)
Supplement: Supplementary file 1 [file nutrients-14-04199-s001.zip › nutrients-1928359-supplementary/Figures S6-S11 (LCMS & NMR).pdf]

Sample Name : C-3071-I-A06 Date Acquired : 22.10.2019 13:18:39 Method File : CortecsC18_50x2,1_40C_Natx_LCMS3.lcm
Injection Volume : 1 Vial# : 6

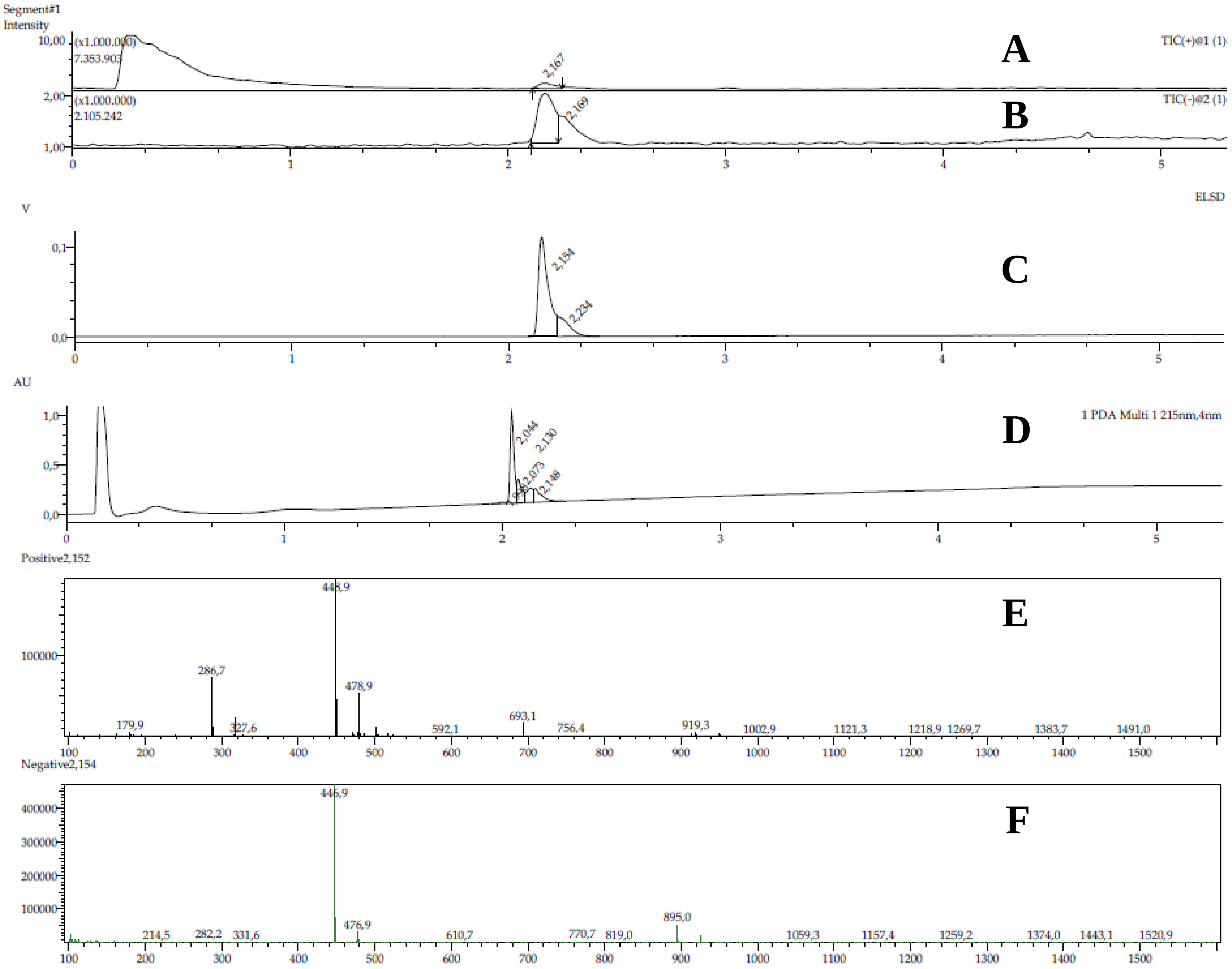

Peak Table C-3071-I-A06

AD2

Peak#	Ret. Time	Area	Area%
1	2,154	347646	81,195
2	2,234	80517	18,805
Total		428162	100,000

PDA Ch1 215nm

Peak#	Ret. Time	Area	Area%
1	1,998	27001	1,222
2	2,044	1160611	52,533
3	2,073	392245	17,754
4	2,130	305785	13,841
5	2,148	323678	14,651
Total		2209319	100,000

Figure S6: HPLC-MS analyses of the fraction C-3071-I-A06 from the *C. chinensis* extract enriched in astragalin. (A) Total ion chromatogram (TIC) in positive ion mode; (B) TIC in negative ion mode; (C) Chromatogram of the evaporative light scattering detector (ELSD); (D) Chromatogram of the photodiode array detector (PDA); (E-F) Mass spectra of the identified compound in positive and negative ion mode.

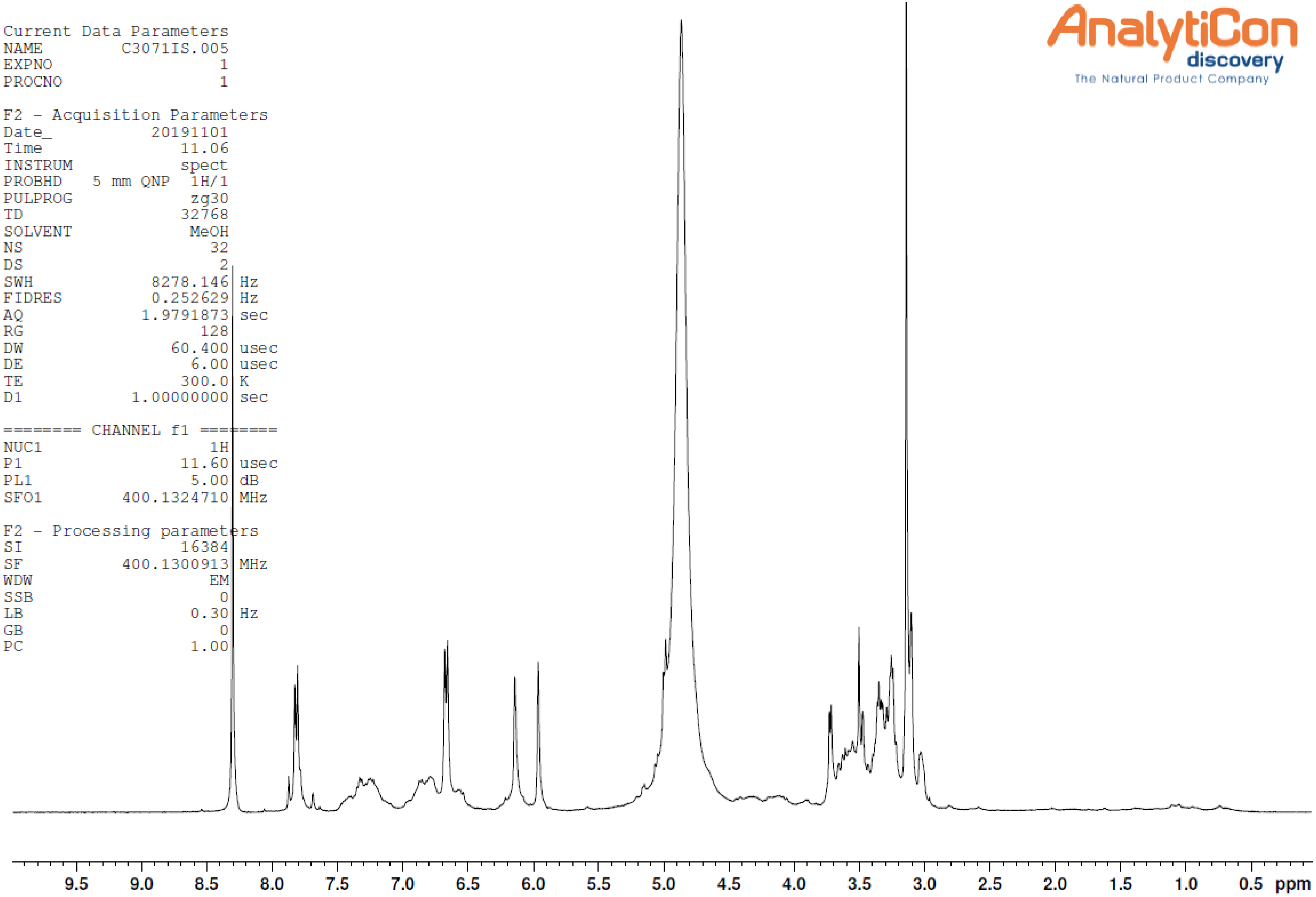

Figure S7: NMR spectrum of astragalin.

Sample Name : C-3071-I-A10 Date Acquired : 22.10.2019 11:51:41 Method File : CortecsC18_50x2,1_40C_Natx_LCMS3.lcm
Injection Volume : 5 Vial# : 10

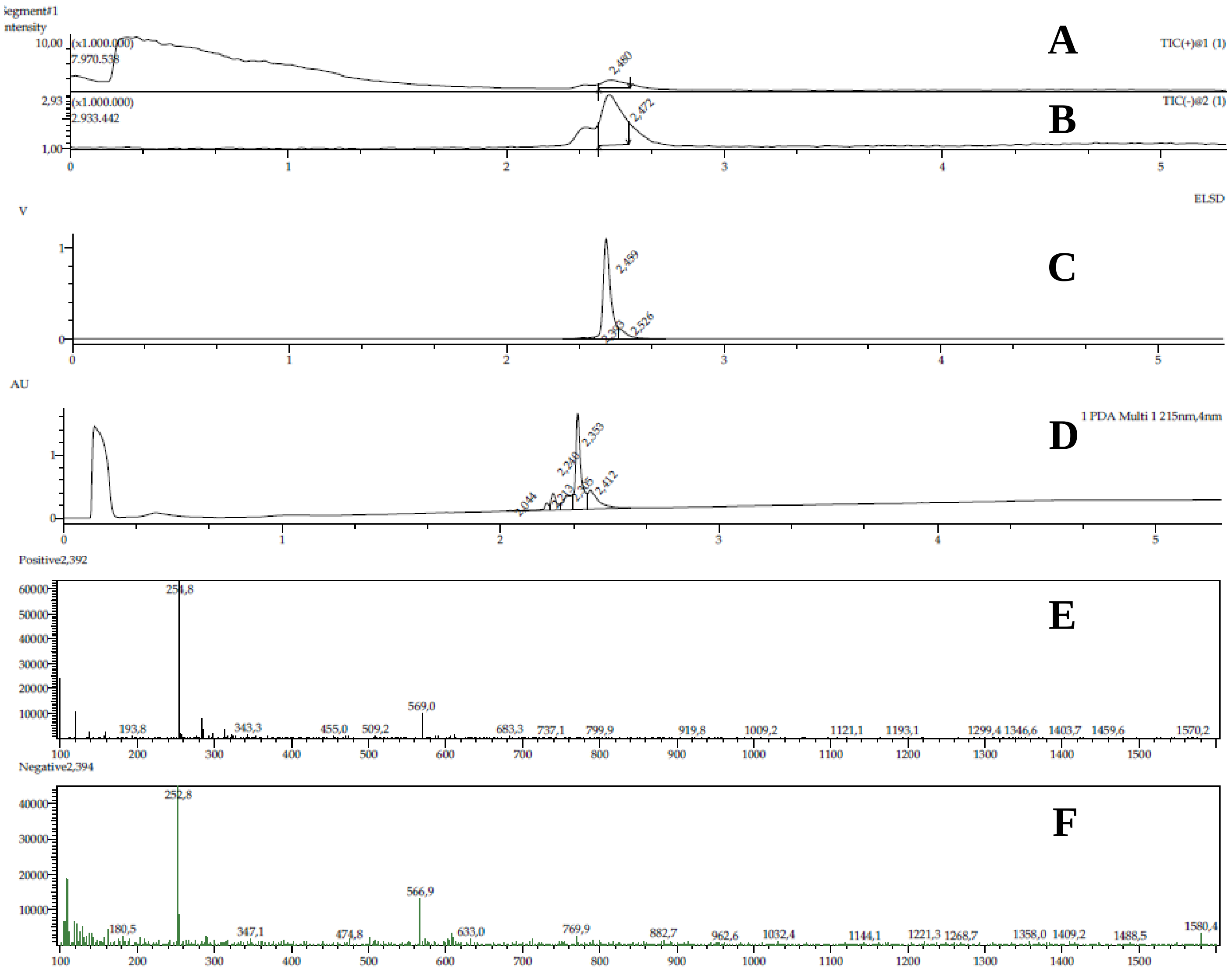

Peak Table C-3071-I-A10

AD2

Peak#	Ret. Time	Area	Area%
1	2,393	50292	1,720
2	2,459	2609445	89,232
3	2,526	264600	9,048
Total		2924338	100,000

PDA Ch1 215nm

Peak#	Ret. Time	Area	Area%
1	2,044	24809	0,513
2	2,213	182719	3,782
3	2,240	454283	9,403
4	2,305	600977	12,439
5	2,353	2666424	55,189
6	2,412	902216	18,674
Total		4831428	100,000

Figure S8: HPLC-MS analyses of the fraction C-3071-I-A10 from the *C. chinensis* extract enriched in pinoresinol. (A) Total ion chromatogram (TIC) in positive ion mode; (B) TIC in negative ion mode; (C) Chromatogram of the evaporative light scattering detector (ELSD); (D) Chromatogram of the photodiode array detector (PDA); (E-F) Mass spectra of the identified compound in positive and negative ion mode.

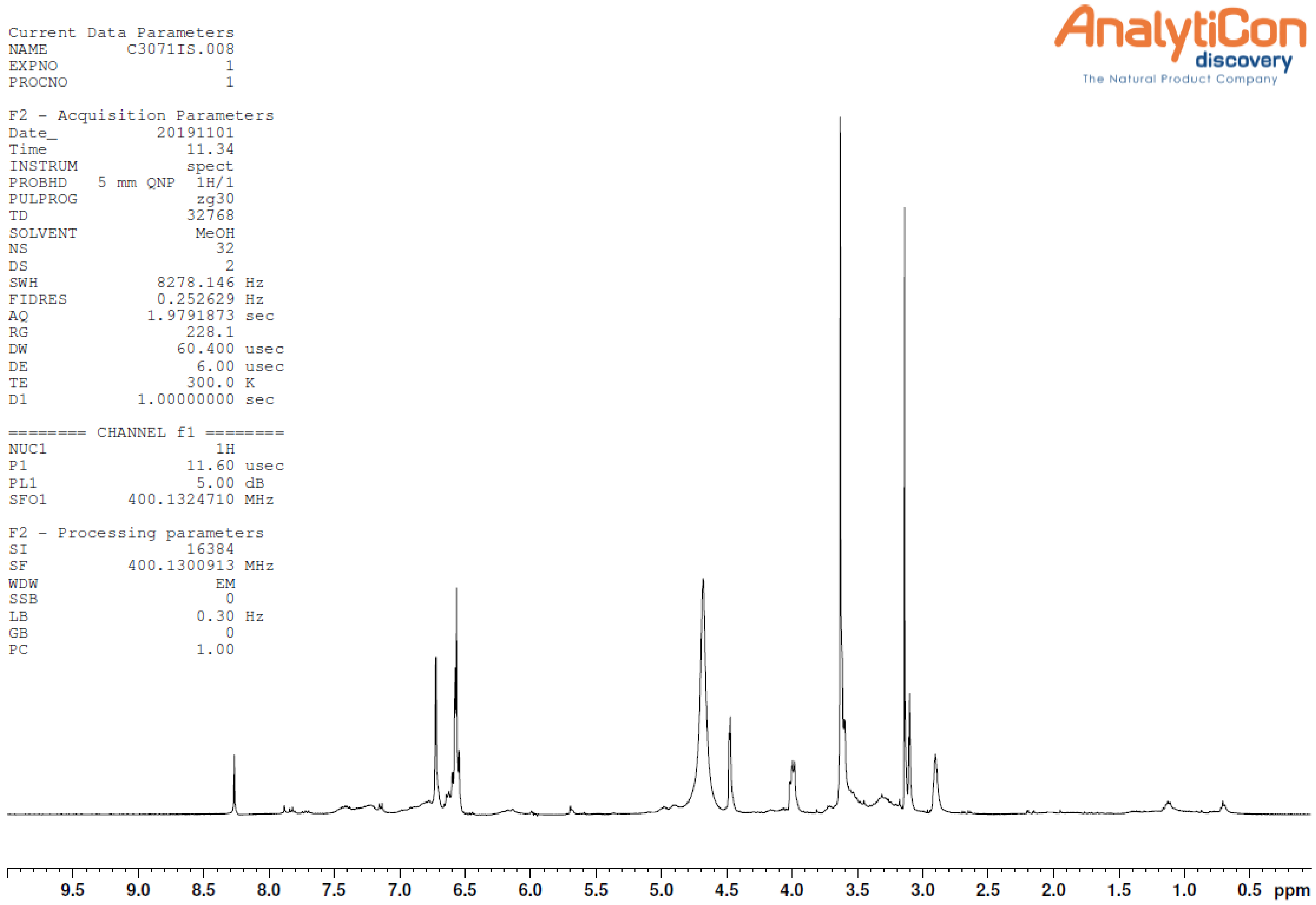

Figure S9: NMR spectrum of pinoresinol.

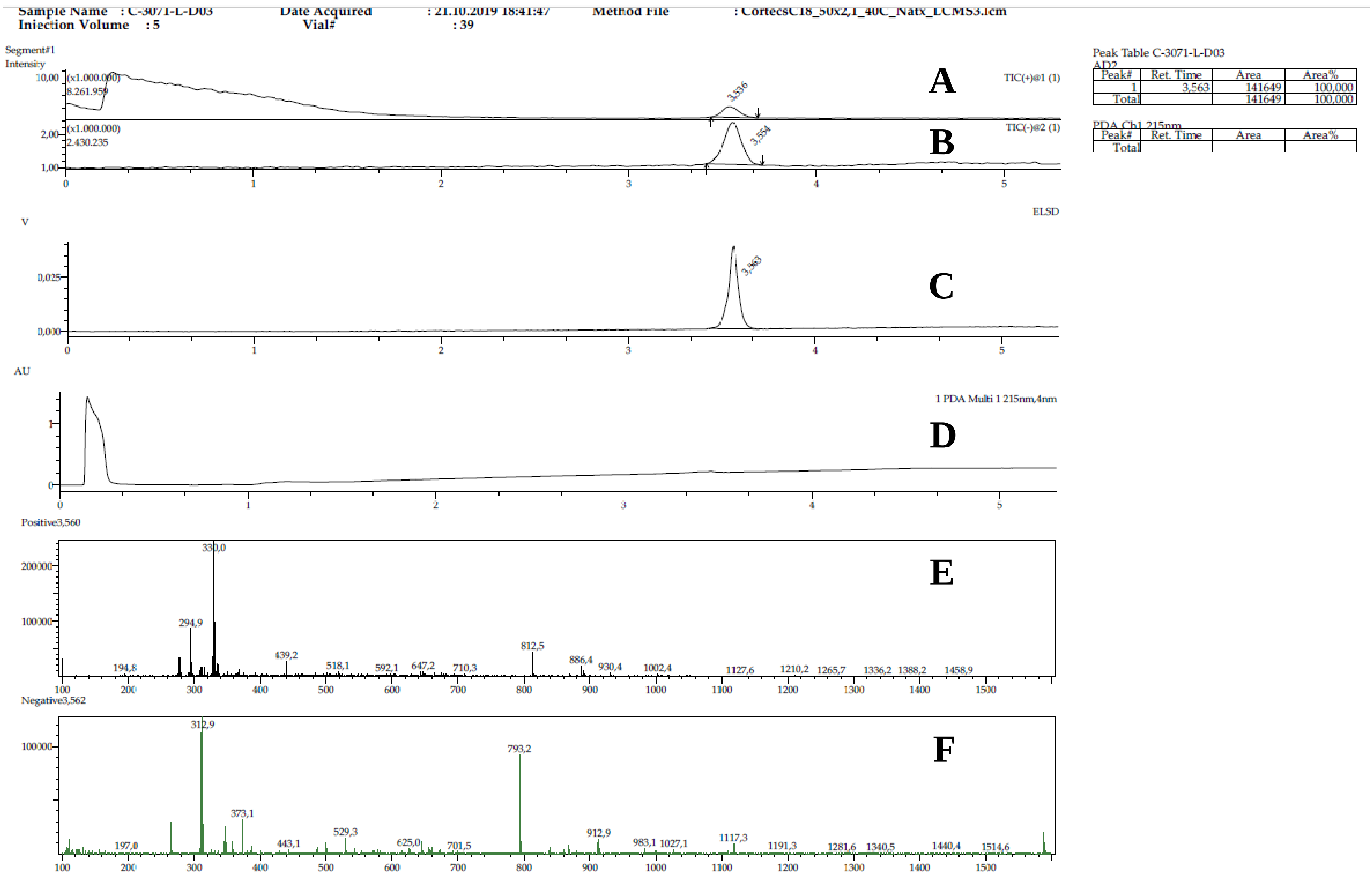

Figure S10: HPLC-MS analyses of the fraction C-3071-L-D03 from the *C. chinensis* extract enriched in zingibroside R1. (A) Total ion chromatogram (TIC) in positive ion mode; (B) TIC in negative ion mode; (C) Chromatogram of the evaporative light scattering detector (ELSD); (D) Chromatogram of the photodiode array detector (PDA); (E-F) Mass spectra of the identified compound in positive and negative ion mode.

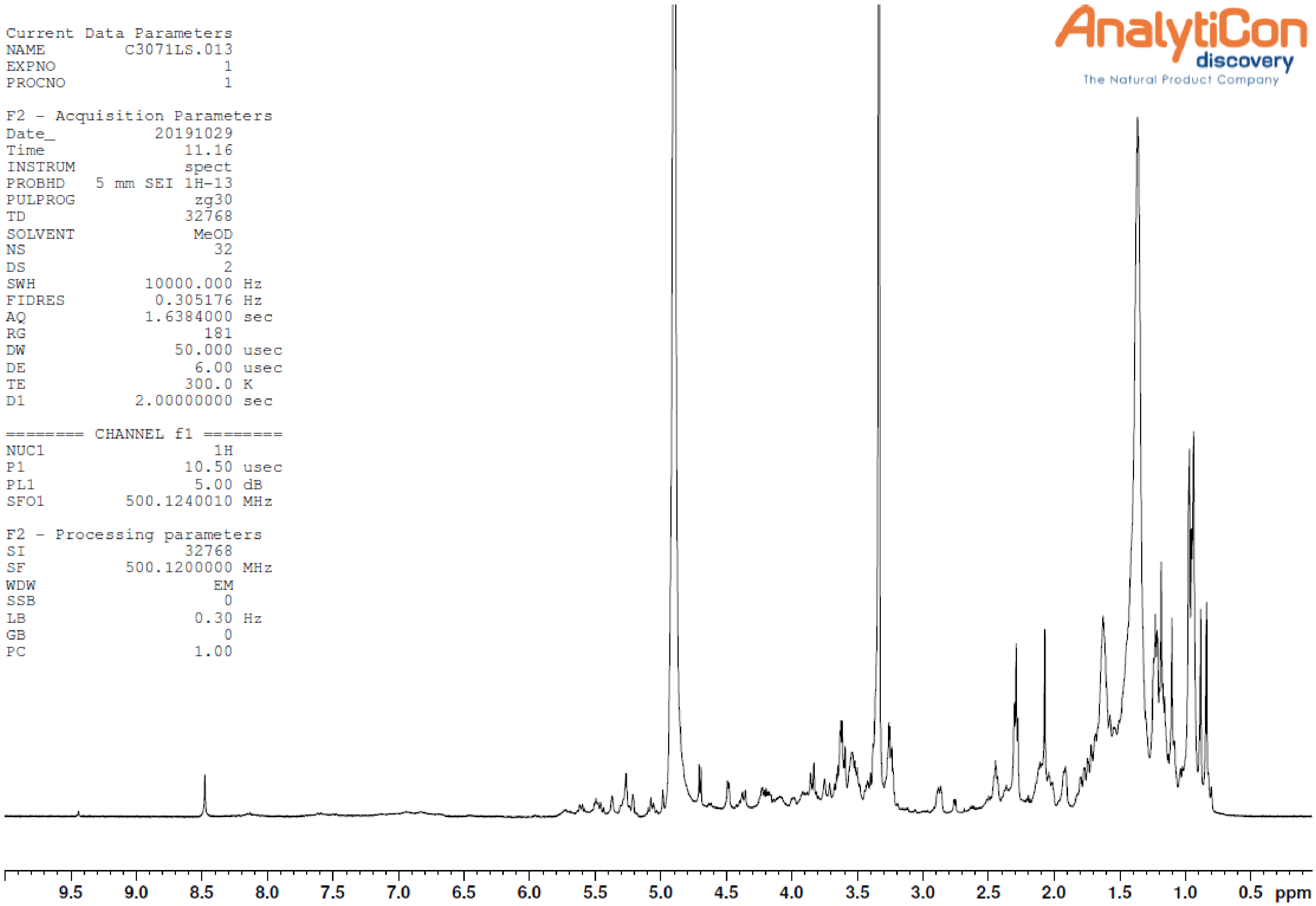

Figure S11: NMR spectrum of zingibroside R1.
