# Supplementary material for: Identification of a Hydroxygallic Acid Derivative, Zingibroside R1 and a Sterol Lipid as Potential Active Ingredients of Cuscuta chinensis Extract That Has Neuroprotective and Antioxidant Effects in Aged Caenorhabditis elegans
Source: Nutrients. 2022 Oct 9;14(19):4199. doi: 10.3390/nu14194199 (PMC9570774; doi:10.3390/nu14194199)
Supplement: Supplementary file 1 [file nutrients-14-04199-s001.zip › nutrients-1928359-supplementary/Figures+S1-S5+(Bioassays+with+fractions).pdf]

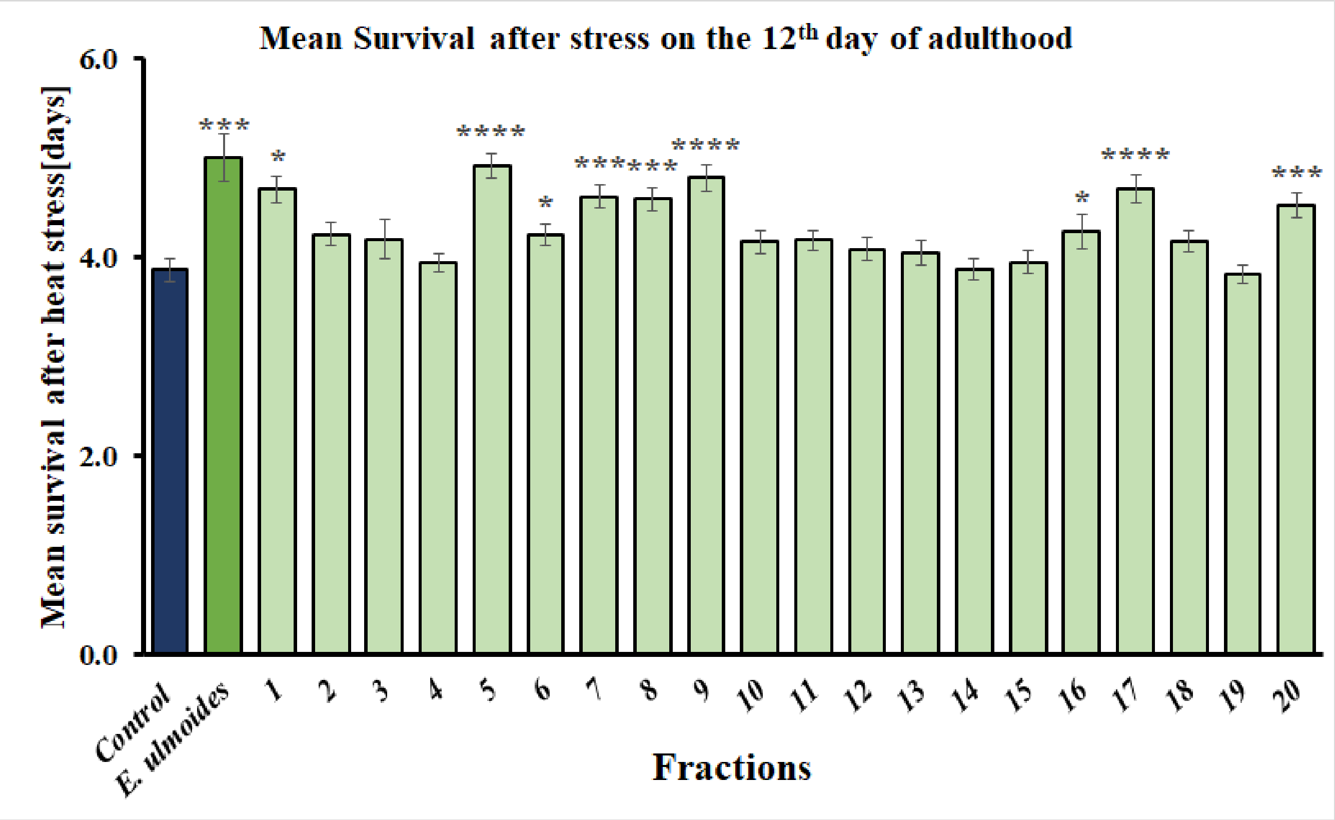

Figure S1: Survival of *C. elegans* treated with 20 fractions of *Eucommia ulmoides* after heat stress exposure (37 °C for 3 h) on the 12th day of adulthood. The mean survival ± SEM after stress exposure is shown from three biological replicates with n ≥ 75-90 worms per treatment. Significant differences were determined by a log-rank test and Bonferroni correction with *p < 0.05; ***p < 0.001 and ****p < 0.0001.

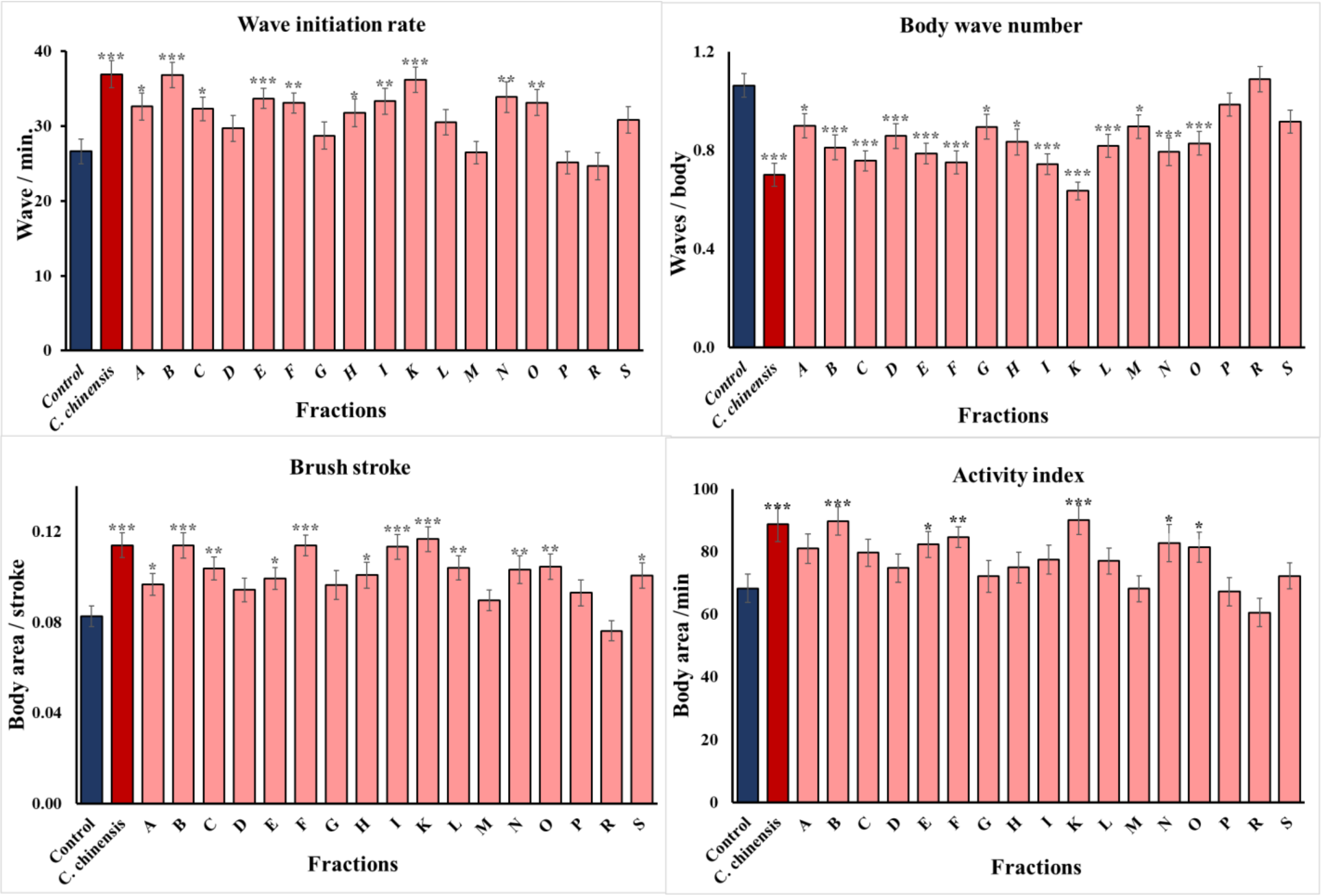

Figure S2: Swim performance of *C. elegans* after treatment with 17 fractions of *C. chinensis*. Body wave number, wave initiation rate, brush stroke and activity index were determined on the 12th day of adulthood. Error bars are the standard error of the mean (SEM) and one bar represents $n \geq 50$ from two independent trials. Statistical significance was determined according to one-way ANOVA and post-hoc Bonferroni test with *($p < 0.05$), ** ($p < 0.01$), or *** ($p < 0.001$).

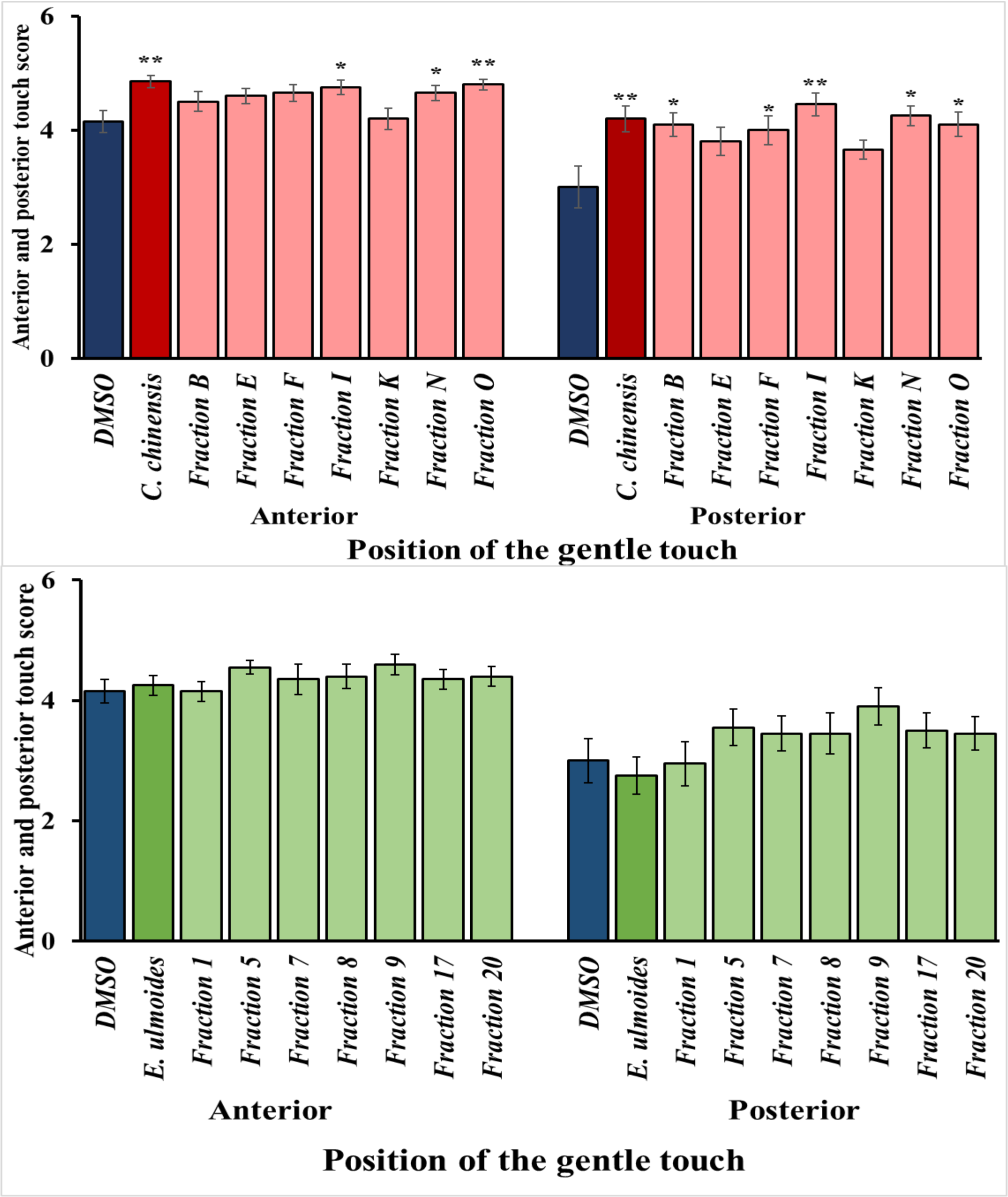

Figure S3: Impact of *C. chinensis* and *E. ulmoides* extracts and selected fractions on the mechanosensory response of *C. elegans* to gentle touches on the 12th day of adulthood. The selection of fractions is based on Figure S1 and S2. The responses after six anterior and six posterior touches were counted for nematodes treated with **(A)** the *C. chinensis* extract and its fractions and **(B)** the *E. ulmoides* extract and its fractions. The rate of behavioral responses was recorded for n≥ 40 nematodes per treatment. The scores were represented as mean ± SEM and significant changes to the control are considered with *($p < 0.05$) or ** ($p < 0.01$) according to one-way ANOVA and post-hoc Bonferroni test.

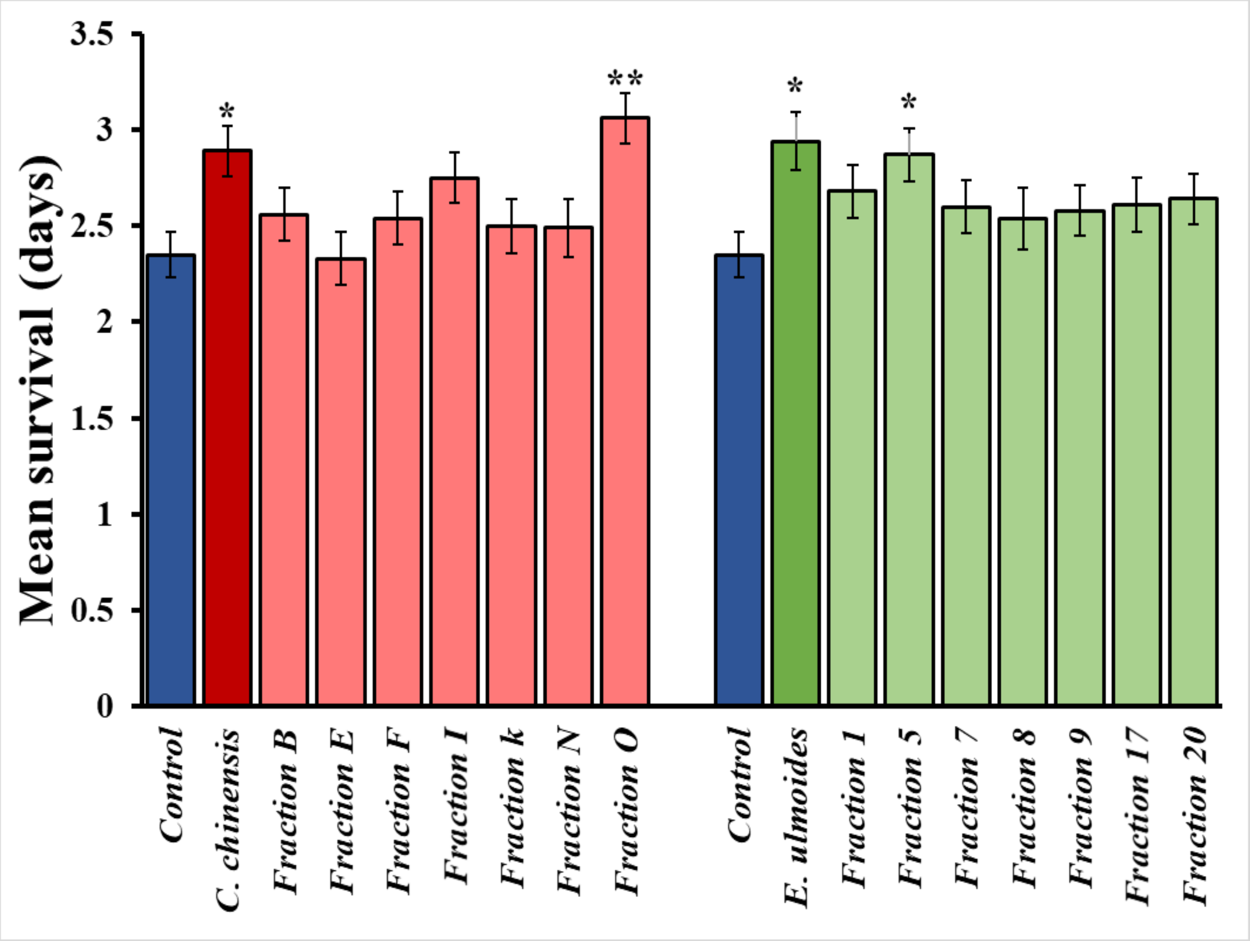

Figure S4: Increase in oxidative stress resistance of *C. elegans* treated with *C. chinensis*, *E. ulmoides* and selected fractions. The selection of fractions is based on Figure S1 and S2. Worms were treated with extracts and fractions starting at the L4 stage, and then exposed to oxidative stress (60 mM paraquat) on the 12th day of adulthood. The mean survival ± SEM from three biological replicates with about 50-60 nematodes per replicate and treatment is shown. Significant differences were determined by a log-rank test and subsequent Bonferroni correction with *p < 0.05 or **p < 0.01.

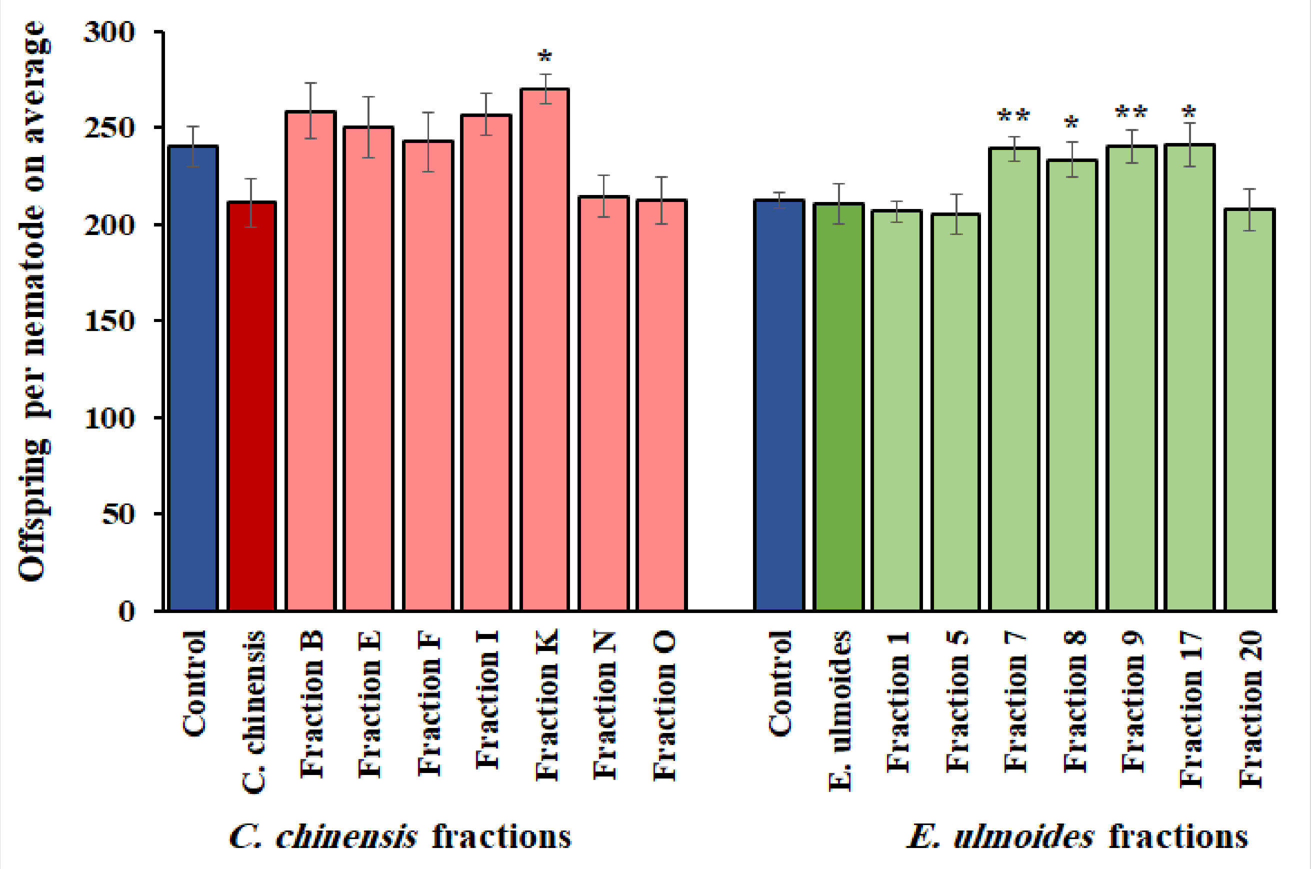

Figure S5: Impact of selected *E. ulmoides* and *C. chinensis* fractions on the reproduction of *C. elegans*. The selection of fractions is based on Figure S1 and S2. The figure shows the mean total offspring per single worm (n = 27–30 nematodes per treatment). The results display the average of three independent experiments, and error bars represent the SEM. Statistically significant differences to the control are indicated with $*p < 0.05$ or $**p < 0.01$according to one-way ANOVA and post-hoc Bonferroni test.
