# Supplementary material for: Identification of a Hydroxygallic Acid Derivative, Zingibroside R1 and a Sterol Lipid as Potential Active Ingredients of Cuscuta chinensis Extract That Has Neuroprotective and Antioxidant Effects in Aged Caenorhabditis elegans
Source: Nutrients. 2022 Oct 9;14(19):4199. doi: 10.3390/nu14194199 (PMC9570774; doi:10.3390/nu14194199)
Supplement: Supplementary file 1 [file nutrients-14-04199-s001.zip › nutrients-1928359-supplementary/File S1 (H2DCFDA-assay).pdf]

Increase of the ROS level found with the H_2DCFDA-assay is rather doubtful

Method: The level of intracellular ROS in living *C. elegans* was measured using 2',7'-dichlorodihydrofluorescein diacetate (H_2DCFDA) as described by Ren, *et al.* [163]. On the 12th day of adulthood, TCM-treated and control worms were transferred to 1.5 mL tubes containing M9 buffer mixed with 10 µM H_2DCFDA, and incubated in the dark for 90 min at 22 °C. After washing twice with M9 buffer, approximately 25 individuals per treatment group were transferred to a 2% agarose pad on a microscope glass slide, and immobilized using 1 M NaN_3. The fluorescence intensity per worm was determined by imaging with a 10x objective using an Axiolab fluorescence microscope (Carl Zeiss, Jena, Germany) with a GFP filter from the Zeiss 4880 series, and equipped with a ProgRes C12 digital camera (Jenoptik, Jena, Germany). Mean fluorescence intensity per individual was quantified densitometrically after splitting the images in the RGB channels using the CellProfiler software [37] combined with bright field images, to determine the body contour. The intensity values were normalized by subtracting the green autofluorescence values measured in extract-treated and control worms of the same age, fed with standard OP50. The normalized intensity represents the ROS level. Four replicates were performed per treatment group.

Results: The dye H_2DCFDA was used to explore the effect of *C. chinensis* and *E. ulmoides* on the ROS level in living wild type worms. H_2DCFDA, a non-fluorescent substance, diffuses into the cells and is oxidized into 2',7'–dichlorofluorescein (DCF) in the presence of ROS, which is a highly fluorescent substance and can be detected by fluorescence spectroscopy. Surprisingly, twelve days-old synchronized *C. elegans,* treated with *C. chinensis* and *E. ulmoides* since the L4 larval stage, showed an increase in fluorescence intensity of 35 % and 25 %, respectively, compared to the DMSO-treated worms (Figure S12).

Discussion: The observed (slight) increase of the ROS level could explain the enhanced stress resistance after *C. chinensis* and *E. ulmoides* treatment according to the (mito)hormesis hypothesis [80,81]. This hypothesis is based on the assumption that the response to mild oxidative stress is beneficial for an organism, which was experimentally underpinned by Schulz, *et al.* [164]. Furthermore, it was shown that antioxidative capacities do not necessarily affect nematode lifespan [165].

However, despite the common use of the H_2DCFDA assay to evaluate ROS levels in *C. elegans* [155], it cannot be considered an accurate method to measure intracellular H_2O_2. First, the assay depends on the uptake of the dye, on the membrane permeability and on diffusion rates into different organs or cell types [13], and we cannot exclude that the extract treatments may change one of these parameters. Furthermore, cytochrome c, transition metals and heme peroxidases can catalyze the oxidation of DCFH to DCF, which itself can produce ROS [166]. Moreover, the H_2DCFDA method is not specific for H_2O_2, since also other ROS can oxidize H_2DCF such as hydroperoxides, hydroxyl radical, and peroxynitrite [13,167]. However, despite the advantages of transgenic strains with an integrated ROS sensor (such as the HyPer strain with a H_2O_2-specific biosensor) over classic chemical sensors, they are also not free of criticism [168]. Another possible method to quantify ROS in *C. elegans* is based on the fluorogenic oxidation of hydroethidine to ethidium [169], which is highly specific for superoxide.

We assume, that the increased ROS level observed in the H_2DCFDA assay was perhaps caused by an increased membrane permeability, leading to an increased intracellular abundance of the dye, and thus, higher fluorescence. This assumption is supported by observations from

Selyutina, *et al.* [170] and [171]. They demonstrated that glycyrrhizic acid, an ingredient of the TCM preparation *Glycyrrhizae Radix et Rhizoma* [172], increases drug solubility, changes the mobility of lipids, forms membrane pores and increases the membrane permeability by about 60%. It is conceivable that ingredients of *E. ulmoides* and *C. chinensis* act in a similar way. Therefore, the increased ROS level is considered rather dubious.

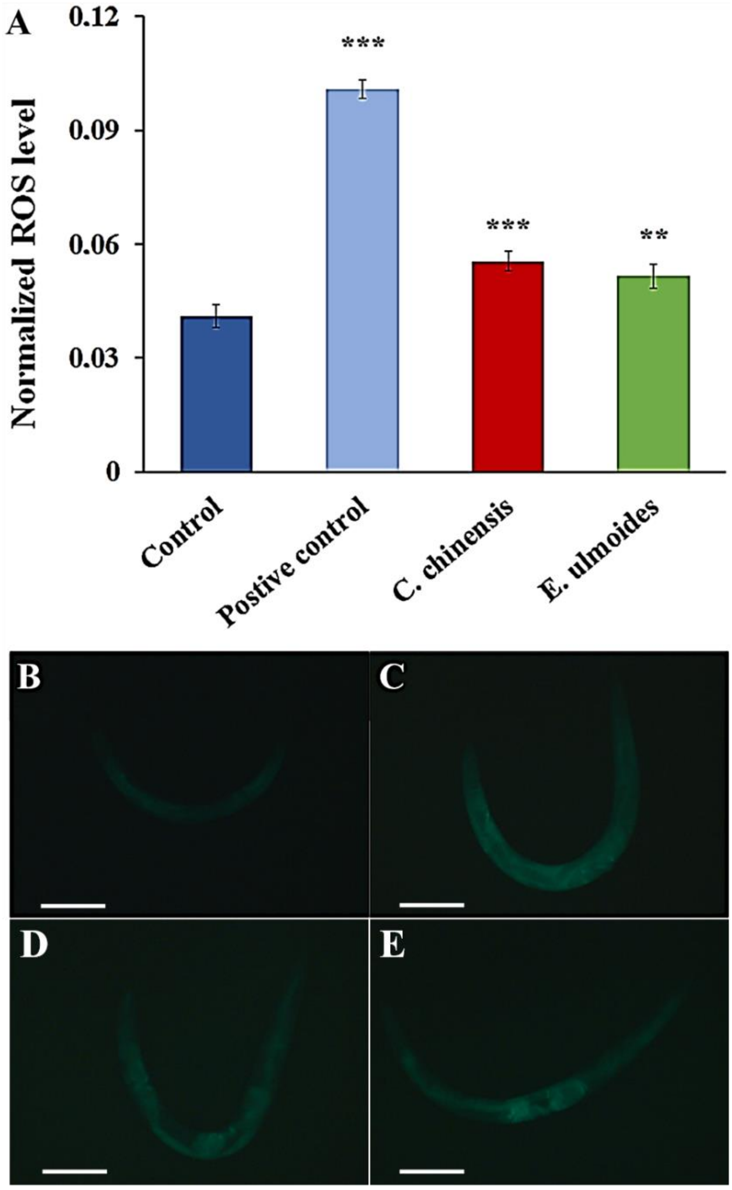

Figure S12: ROS level in *C. elegans* on the 12th day of adulthood treated with *C. chinensis* and *E. ulmoides*. **(A)** The mean normalized fluorescence intensities are shown for wild-type *C. elegans* exposed to 10 µM H_2DCFDA on the 12th day of adulthood. Error bars represent the standard error of the mean (SEM) and significant differences to the control are considered with ** ($p < 0.01$) and *** ($p < 0.001$) according to one-way ANOVA and post-hoc Bonferroni test. **(B-E)** Representative images show the fluorescence intensity levels in untreated worms before **(B)** and after **(C)** applying of 10 mM H_2O_2 (positive control) as well as in *C. cuscuta* **(D)** and *E. ulmoides* **(E)** treated nematodes. The white scale bars represent 200 µm.

References

13. Labuschagne, C.F.; Brenkman, A.B. Current methods in quantifying ros and oxidative damage in *caenorhabditis elegans* and other model organism of aging. *Ageing Res. Rev.* **2013**, *12*, 918–930.
37. McQuin, C.; Goodman, A.; Chernyshev, V.; Kamentsky, L.; Cimini, B.A.; Karhohs, K.W.; Doan, M.; Ding, L.; Rafelski, S.M.; Thirstrup, D. Cellprofiler 3.0: Next-generation image processing for biology. *PLoS Biol.* **2018**, *16*, e2005970.
80. Ristow, M. Unraveling the truth about antioxidants: Mitohormesis explains ros-induced health benefits. *Nat. Med.* **2014**, *20*, 709–711.
81. Ristow, M.; Schmeisser, K. Mitohormesis: Promoting health and lifespan by increased levels of reactive oxygen species (ros). *Dose Response* **2014**, *12*, 288–341.
155. Saul, N.; Möller, S.; Cirulli, F.; Berry, A.; Luyten, W.; Fuellen, G. Health and longevity studies in *C. elegans*: The "healthy worm database" reveals strengths, weaknesses and gaps of test compound-based studies. *Biogerontology* **2021**, *22*, 215–236.
163. Ren, T.; Zhang, H.; Wang, J.; Zhu, J.; Jin, M.; Wu, Y.; Guo, X.; Ji, L.; Huang, Q.; Yang, H., *et al.* Mcu-dependent mitochondrial ca^{2+} inhibits nad^{+}/sirt3/sod2 pathway to promote ros production and metastasis of hcc cells. *Oncogene* **2017**, *36*, 5897-5909.
164. Schulz, T.J.; Zarse, K.; Voigt, A.; Urban, N.; Birringer, M.; Ristow, M. Glucose restriction extends *Caenorhabditis elegans* life span by inducing mitochondrial respiration and increasing oxidative stress. *Cell Metab.* **2007**, *6*, 280-293.
165. Pun, P.B.; Gruber, J.; Tang, S.Y.; Schaffer, S.; Ong, R.L.; Fong, S.; Ng, L.F.; Cheah, I.; Halliwell, B. Ageing in nematodes: Do antioxidants extend lifespan in *Caenorhabditis elegans*? *Biogerontology* **2010**, *11*, 17-30.
166. Dikalov, S.I.; Harrison, D.G. Methods for detection of mitochondrial and cellular reactive oxygen species. *Antioxidants & redox signaling* **2014**, *20*, 372-382.
167. Yang, C.; Jiang, L.; Zhang, H.; Shimoda, L.A.; DeBerardinis, R.J.; Semenza, G.L. Analysis of hypoxia-induced metabolic reprogramming. *Methods enzymol.* **2014**, 542, 425-455.
168. Braeckman, B.P.; Smolders, A.; Back, P.; De Henau, S. In vivo detection of reactive oxygen species and redox status in *Caenorhabditis elegans*. *Antioxid Redox Signal* **2016**, *25*, 577-592.
169. Schlotterer, A.; Hamann, A.; Kukudov, G.; Ibrahim, Y.; Heckmann, B.; Bozorgmehr, F.; Pfeiffer, M.; Hutter, H.; Stern, D.; Du, X., *et al.* Apurinic/apyrimidinic endonuclease 1, p53, and thioredoxin are linked in control of aging in *C. elegans*. *Aging Cell* **2010**, *9*, 420-432.
170. Selyutina, O.Y.; Apanasenko, I.E.; Kim, A.V.; Shelepova, E.A.; Khalikov, S.S.; Polyakov, N.E. Spectroscopic and molecular dynamics characterization of glycyrrhizin membrane-modifying activity. *Colloids Surf B Biointerfaces* **2016**, *147*, 459-466.
171. Selyutina, O.Y.; Polyakov, N.E.; Korneev, D.V.; Zaitsev, B.N. Influence of glycyrrhizin on permeability and elasticity of cell membrane: Perspectives for drugs delivery. *Drug Deliv* **2016**, *23*, 858-865.
172. Li, N.; Zhou, T.; Wu, F.; Wang, R.; Zhao, Q.; Zhang, J.Q.; Yang, B.C.; Ma, B.L. Pharmacokinetic mechanisms underlying the detoxification effect of glycyrrhizae radix et rhizoma (gancao): Drug metabolizing enzymes, transporters, and beyond. *Expert Opin. Drug Metab. Toxicol.* **2019**, *15*, 167-177.
